# Gender-Dependent Characteristics of Serum 1,25-Dihydroxyvitamin D/25-Hydroxyvitamin D Ratio for the Assessment of Bone Metabolism

**DOI:** 10.7759/cureus.18070

**Published:** 2021-09-18

**Authors:** Manami Fujita-Yamashita, Koichiro Yamamoto, Hiroyuki Honda, Yoshihisa Hanayama, Kazuki Tokumasu, Yasuhiro Nakano, Kou Hasegawa, Hideharu Hagiya, Mikako Obika, Hiroko Ogawa, Fumio Otsuka

**Affiliations:** 1 Department of General Medicine, Okayama University Graduate School of Medicine, Dentistry and Pharmaceutical Sciences, Okayama, JPN

**Keywords:** aging, bone metabolism, calcium, cyp27b1, vitamin d

## Abstract

Objectives

Vitamin D deficiency, which is common worldwide, increases the risks of falls and fractures and can lead to increased morbidity and mortality. However, the clinical utility and relevance of vitamin D activation remain unknown. The aim of the present study was to clarify the clinical usefulness of serum 1,25-dihydroxyvitamin D (1,25D)/25-hydroxyvitamin D (25D) ratio for assessment of the extent of bone metabolism.

Methods

We retrospectively screened data for 87 patients whose serum 1,25D and 25D levels were measured. Eight patients who were taking vitamin D preparations were excluded, and data for 79 patients (33 males and 46 females) were analyzed. Since menopausal status can be associated with serum vitamin D level, we divided the patients by gender and divided the female patients into two groups at the age of 50 years.

Results

The median serum 1,25D/25D ratio was significantly lower in males than in females, with the most considerable difference in all males [4.1 (interquartile range: 2.3-5.8) × 10^−3^] versus elderly females (aged ≧50 years) [7.9 (3.3-10.1) × 10^−3^). Main disorders were endocrine (30.6%), inflammatory (18.5%), and bone-related (16.7%) disorders. The ratios of serum 1,25D/25D had significant negative correlations with femoral dual-energy X-ray absorptiometry % young adult mean (DEXA %YAM) (*R=*−0.35) and lumbar DEXA %YAM (*R=*−0.32). Significant correlations were found between the 1,25D/25D ratio and serum levels of inorganic phosphate (iP), parathyroid hormone, and alkaline phosphatase (ALP). The 1,25D/25D ratio had gender-specific characteristics: the ratio was significantly correlated with age in males (*R=*−0.49), while it was significantly correlated with BMI in females (*R=*0.34).

Conclusions

The results of this study suggested that vitamin D activity is negatively correlated with bone mineral density, being reduced in aged males but enhanced in obese females.

## Introduction

Vitamin D is obtained in the body by food intake or by production from 7-dehydrocholesterol by exposure of the skin to ultraviolet B radiation [1]. Vitamin D is first metabolized in the liver to 25-hydroxyvitamin D (25D), which is a major circulating metabolite [1]. In the kidney, 25D is subsequently metabolized to the hormonally active form, 1,25-dihydroxyvitamin D (1,25D), via 1α-hydroxylase encoded by the CYP27B1 gene [1]. Renal CYP27B1 is regulated primarily by parathyroid hormone (PTH) and calcitonin in stimulatory manners and by fibroblast growth factor-23 (FGF-23) and 1,25D itself in inhibitory manners [1]. CYP27B1 also exists in extra-renal sites such as macrophages, and its expression in extra-renal sites is associated with granuloma-forming disorders and is regulated mainly by type I and type II interferons (IFNs) [2]. 1,25D has a cellular effect through the vitamin D receptor (VDR) [3], which leads to calcium absorption in the gut, bone metabolism, and parathyroid function.

Serum 25D level has been considered to be a reliable marker of vitamin D status: serum 25D level below 20 ng/mL is defined as vitamin D deficiency and serum 25D level below 30 ng/mL is defined as vitamin D insufficiency [4]. A recent study showed that a low vitamin D status is common worldwide and is associated with various diseases including kidney, heart, and liver failure, secondary hyperparathyroidism, osteomalacia, inflammatory bowel disease, granuloma-forming disorders (sarcoidosis and tuberculosis), and cancer [5]. Vitamin D deficiency also increases the risks of falls, fractures, bone loss, and sarcopenia [6-8], leading to worse outcomes of illness severity, morbidity, and mortality [9-11]. However, since the evaluation of only serum 25D level did not explain hormonal activity of vitamin D, it is conceivable that we should take serum 1,25D level into account as well. The clinical utility of and the relevance to pathophysiology of evaluation of the ratio 1,25D/25D, which indicates the extent of vitamin D activation, have remained unknown.

In the present study, we retrospectively investigated the relevance of activation of vitamin D to various clinical characteristics of patients who visited a general medicine department.

## Materials and methods

Study design

We conducted a single-center cross-sectional study: the medical records of 87 patients (male/female: 35/52), whose serum levels of 25D and 1,25D were measured between January 2017 and December 2019 at the Department of General Medicine, Okayama University Hospital were screened. Of those patients, 8 patients (2 males and 6 females) who were taking vitamin D preparations were excluded, and data for 79 patients (33 males (41.8%) and 46 females (58.2%)) were analyzed. The decision to examine serum levels of 25D or 1,25D had been made individually by physicians for clinical purposes when vitamin D-related disorders such as hyper- and hypoparathyroidism, osteoporosis, and granuloma-forming disorders were suspected [5]. Data for other biochemical parameters were obtained within one week from the measurement of 25D or 1,25D. The present study was approved by the Ethical Committee of Okayama University Hospital (KEN-2001-022) and adhered to the Declaration of Helsinki.

Analysis of clinical parameters

Information on the patients’ main disorders and past medical histories was obtained from hospital medical records. Information on age, gender, race, body mass index (BMI), and self-rating depression scale (SDS) was also obtained [12]. Information on the following biochemical parameters was also obtained: white blood cells, red blood cells, hemoglobin, hematocrit and platelets for blood cell counts; 25D, 1,25D, calcium (Ca), corrected Ca (cCa), inorganic phosphate (iP), cCa × iP, alkaline phosphatase (ALP), bone-specific alkaline phosphatase (BAP), and intact PTH for bone metabolism; total bilirubin, total protein, albumin, aspartate aminotransferase (AST), alanine aminotransferase (ALT), lactate dehydrogenase (LDH), γ-glutamyl transpeptidase (γGTP), sodium, potassium, chloride, magnesium, blood urea nitrogen (BUN), creatinine and estimated glomerular filtration rate (eGFR) for liver and renal functions; prothorombin time-international normalized ratio (PT-INR), activated partial thromboplastin time (APTT), and d-dimer for coagulatory markers; C-reactive protein (CRP), erythrocyte sedimentation rate in one hour (ESR), ferritin, 50% hemolytic unit of complement (CH50) and angiotensin-converting enzyme (ACE) for inflammatory markers; and hemoglobin A1c (HbA1c), plasma glucose, total cholesterol, uric acid, adrenocorticotropic hormone (ACTH), cortisol, prolactin, luteinizing hormone (LH), follicle-stimulating hormone (FSH), growth hormone (GH), thyroid-stimulating hormone (TSH), free thyroxine (FT4), ratio of TSH/FT4, and total testosterone for endocrine and metabolic markers. The levels of those parameters were determined by using an auto-analyzer system at the Central Laboratory of Okayama University Hospital. The levels of 25D and 1,25D were determined by a radioimmunoassay and a chemiluminescent immunoassay, respectively, at LSI Medience Corporation (Tokyo). Radiological data of dual-energy X-ray absorptiometry % young adult mean (DEXA %YAM) in the femoral bone and lumbar spine were evaluated as we previously reported [13].

Statistical analysis

All statistical analyses were performed using EZR, version 1.40 (Saitama Medical Center, Jichi Medical University, Saitama, Japan), which is a graphical user interface for R (The R Foundation for Statistical Computing, Vienna, Austria) [14]. In more detail, it is a modified version from R commander designed to add functions of frequently used statistics in biostatistics. Continuous measurements were statistically tested using the Mann-Whitney U test, Spearman's rank correlation coefficient, or Kruskal-Wallis test. The Mann-Whitney U test and Spearman's rank correlation coefficient were treated as two-sided. When differences were detected by the Kruskal-Wallis test, the Steel-Dwass post-hoc test was used for further analysis between the groups. *P*-values less than 0.05 were regarded as statistically significant. There were potential confounding factors: diseases such as hyperparathyroidism, sarcoidosis, inflammatory bowel disease, osteoporosis, and osteomalacia or rickets; environmental factors such as seasonal changes, lifestyles related to sunlight exposure, and nutritional intake; and gender and the human race. Since menopause has been reported to cause changes in serum vitamin D level and bone metabolism [15], we conducted stratified analysis by gender and we divided the female patients into two groups at the age of 50 years [16].

## Results

Patients’ characteristics and relations to vitamin D activity

**Figure 1:**
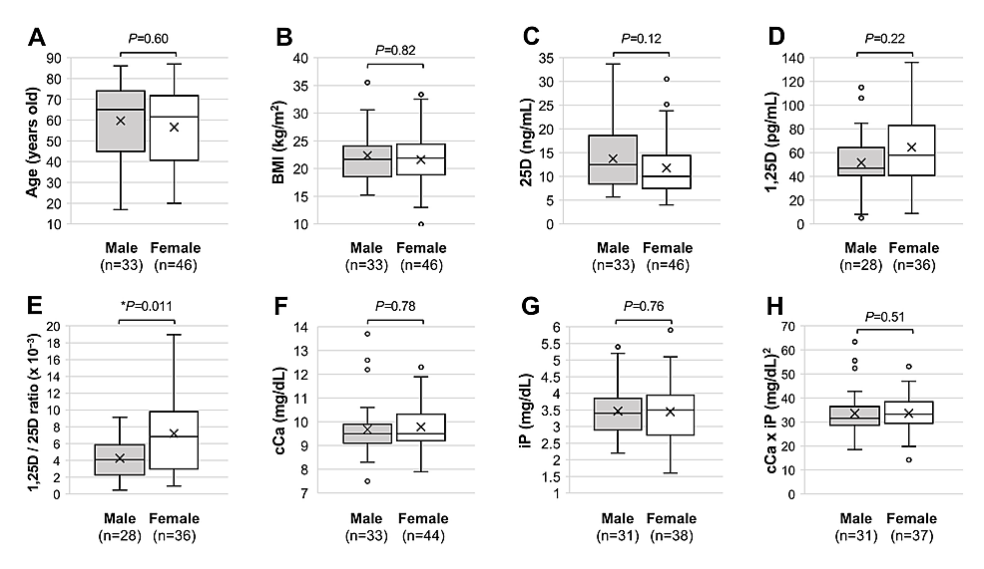
Characteristics of the male and female patients Age (A), BMI (B) and serum levels of 25D (C), 1,25D (D), 1,25D/25D ratio (E), cCa (F), iP (G), and cCa × iP (H) are shown. In each panel, the upper horizontal line, lower horizontal line, and horizontal bar of the box indicate the 75th percentile, 25th percentile, and median, respectively. The horizontal bars outside the box are the maximum and minimum values within 1.5 times the interquartile range. The X sign within the box indicates the mean. *P<0.05, a statistically significant difference between the indicated groups (A-H). BMI: body mass index; cCa: corrected calcium; iP: inorganic phosphate; n.s.: not significant; 1,25D: 1,25-dihydroxyvitamin D; 25D: 25-hydroxyvitamin D.

The 79 patients analyzed in the present study included 33 males (41.8%) and 46 females (58.2%). As shown in Figure 1A, the median age of the male patients was 65 [interquartile range (IQR): 45-74] years and that of the female patients was 61.5 (IQR: 40.8-71.8) years, and there was no significant difference in the median ages. The median BMI of males was 21.7 (IQR: 18.6-24.1) kg/m^2^ and that of females was 22.0 (IQR: 18.9-24.5) kg/m^2^, and the difference was not significant (Figure 1B). The median serum 25D level in males was 12.5 (IQR: 8.4-18.6) ng/mL and that in females was 10 (IQR: 7.5-14.5) ng/mL (Figure 1C). The median serum 1,25D level in males was 47 (IQR: 41.0-64.3) pg/mL and that in females was 58 (IQR: 41.0-83.0) pg/mL (Figure 1D). There was no significant difference between males and females in the level of 25D (Figure 1C) or 1,25D (Figure 1D), but the median serum 1,25D/25D ratio was significantly lower in males than in females: the median ratio in males was 4.1 (IQR: 2.3-5.8) × 10^−3^ and that in females was 6.8 (IQR: 3.0-9.8) × 10^−3^ (Figure 1E). Regarding electrolytes, serum cCa, iP, and cCa × iP levels were not significantly different in males and females (Figure 1F-1H). The median serum levels of cCa were 9.5 (IQR: 9.1-9.9) mg/dL in males and 9.5 (IQR: 9.2-10.3) mg/mL in females, those of iP were 3.4 (IQR: 2.9-3.9) mg/dL in males and 3.5 (IQR: 2.8-4.0) mg/dL in females, and those of cCa × iP were 31.5 (IQR: 28.6-36.5) (mg/dL)^2^ in males and 33.3 (IQR: 29.5-38.4) (mg/dL)^2^ in females.

The patients' main disorders and past medical histories are shown in Table 1. The most frequent main disorders were endocrine disorders (30.6%, 33 of 108 disorders) including primary hyperparathyroidism (10.2%, 11/108) and ectopic hyperparathyroidism (2.9%, 2/108) for vitamin D-related disorders followed by inflammatory disorders (18.5%, 20/108) including sarcoidosis (2.8%, 3/108) and inflammatory bowel disease (2.8%, 3/108) and bone-related disorders including osteoporosis (13.9%, 15/108) and osteomalacia or rickets (2.8%, 3/108) (Table 1, left). The most frequent past medical history was malignancy (26.8%, 19 of 71 histories) followed by bone-related diseases (16.9%, 12/71) including fractures (11.3%, 8/71) and osteoporosis (5.6%, 4/71), metabolic diseases (14.1%, 10/71), and ureterolithiasis (11.3%, 8/71) (Table 1, right). Past medical histories possibly related to vitamin D deficiency included sarcoidosis (1.4%, 1/71) in inflammatory diseases (8.5%, 6/71), while there was no past history of hyper- or hypoparathyroidism (Table 1, right).

**Table 1:** Main disorders and past medical histories CNS: central nervous system.

Main disorders		Past medical histories	
Categories	Total (%)	Categories	Total (%)
Endocrine disorder	33 (30.6)	Malignancy	19 (26.8)
Inflammatory disorder	20 (18.5)	Bone-related disease	12 (16.9)
Bone-related disorder	18 (16.7)	Metabolic disease	10 (14.1)
Mental disorders	10 (9.3)	Ureterolithiasis	8 (11.3)
Gastroenterological	9 (8.3)	Inflammatory disease	6 (8.5)
Bacterial infection	6 (5.6)	CNS-related disease	5 (7.0)
Haematological	4 (3.7)	Mental disease	5 (7.0)
Malignancy	2 (1.9)	Endocrine disease	4 (5.6)
Other	6 (5.6)	Other	2 (2.8)
Total	108 (100)	Total	71 (100)

Gender-dependent differences of vitamin D activity and bone mineral density

**Figure 2:**
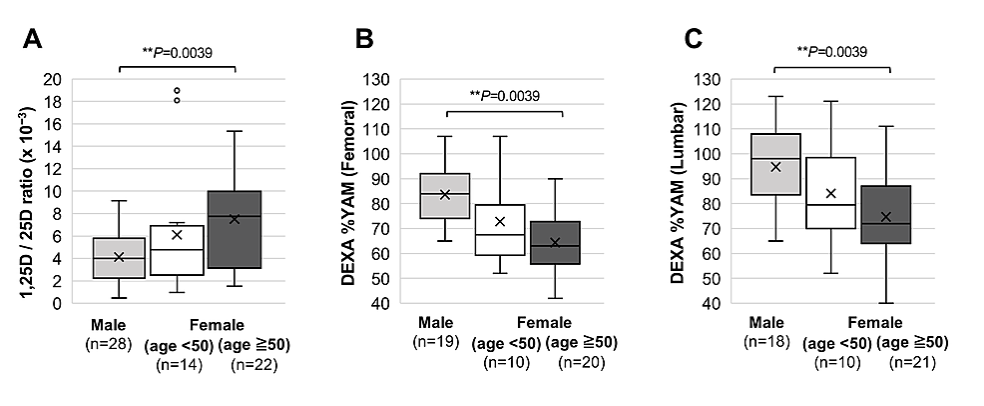
Gender-dependent differences of serum 1,25D/25D ratio and bone mineral densities Serum 1,25D/25D ratio (A) and bone mineral densities in the femoral bone (B) and lumbar spine (C) were compared between male patients, female patients aged <50 years, and female patients aged ≧50 years. The details of each panel are shown in the legend of Figure 1. **P<0.01 and *P<0.05, statistically significant correlations between the indicated factors. 1,25D: 1,25-dihydroxyvitamin D; 25D: 25-hydroxyvitamin D; DEXA %YAM: dual-energy X-ray absorptiometry % young adult mean.

Since menopausal status has been reported to be associated with vitamin D and bone metabolism [15], we divided the female patients into two groups at the age of 50 years [16]. Serum 1,25D/25D ratios were found to be significantly higher in female patients aged ≧50 years (median ratio, 7.9 (IQR: 3.3-10.1) × 10^−3^) than in male patients (median ratio, 4.1 (IQR: 2.3-5.8) × 10^−3^) (Figure 2A). Regarding bone mineral density, female patients aged ≧50 years had significantly lower values of DEXA %YAM in both the femoral bone (Figure 2B) and lumbar spine (Figure 2C) than those in male patients. The median values of DEXA %YAM in male patients were 84% (IQR: 74-92%) in the femoral bone and 98% (IQR: 83.5-108%) in the lumbar spine, and those in female patients aged ≧50 years were 63% (IQR: 55.8-72.8%) in the femoral bone and 72% (IQR: 64-87%) in the lumbar spine.

Relationships of serum 1,25D/25D ratios with clinical markers in bone metabolism

**Figure 3:**
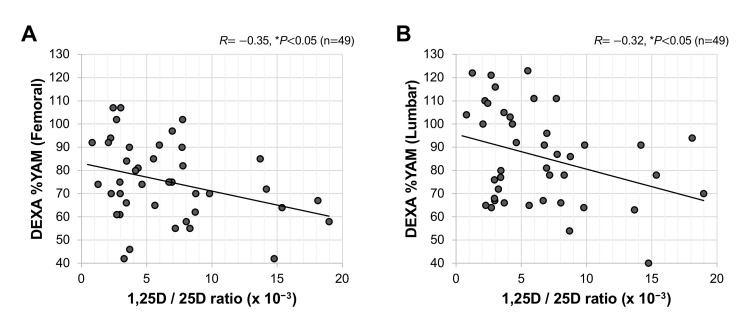
Correlations of serum 1,25D/25D ratio with bone mineral densities Correlations of femoral DEXA %YAM (A) and lumbar DEXA %YAM (B) with a 1,25D/25D ratio in all patients are shown. *P<0.05, statistically significant correlations between the indicated factors (A, B). 1,25D: 1,25-dihydroxyvitamin D; 25D: 25-hydroxyvitamin D; DEXA %YAM: dual-energy X-ray absorptiometry % young adult mean.

**Figure 4:**
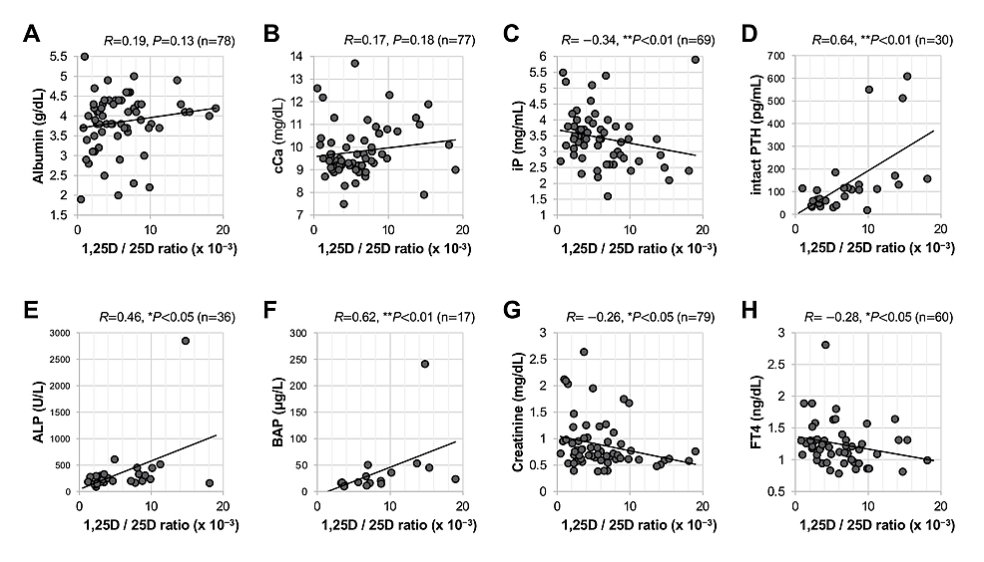
Relationships of 1,25D/25D ratio with bone metabolism and clinical characteristics Correlations of serum 1,25D/25D ratios with albumin (A), cCa (B), iP (C), intact PTH (D), ALP (E), BAP (F), creatinine (G), and FT4 (H) are shown. **P<0.01 and *P<0.05, statistically significant correlations between the indicated factors. ALP: alkaline phosphatase; BAP: bone-specific alkaline phosphatase; cCa: corrected calcium; FT4: free thyroxine; iP: inorganic phosphate; n.s.: not significant; 1,25D: 1,25-dihydroxyvitamin D; PTH: parathyroid hormone; 25D: 25-hydroxyvitamin D.

We investigated the correlations of serum 1,25D/25D ratios with various clinical parameters. Of note, as shown in Figure 3, 1,25D/25D ratios had significant negative correlations with bone mineral densities including femoral DEXA %YAM (*R*=−0.35, *P<0.05; Figure 3A) and lumbar DEXA %YAM (*R*=−0.32, *P<0.05; Figure 3B). Regarding biochemical markers of bone metabolism, 1,25D/25D ratios were not correlated with serum albumin (*R*=0.19, *P*=0.13; Figure 4A) and cCa (*R*=0.17, *P*=0.18; Figure 4B) levels. On the other hand, 1,25D/25D ratios had significant positive correlations with serum levels of iP (*R*=−0.34, **P<0.01; Figure 4C), intact PTH (*R*=0.64, **P<0.01; Figure 4D), ALP (*R*=0.46, *P<0.05; Figure 4E), and BAP (*R*=0.62, **P<0.01; Figure 4F). 1,25D/25D ratios had significant negative correlations with creatinine (*R*=−0.26, *P<0.05; Figure 4G) and FT4 (*R*=−0.28, *P<0.05; Figure 4H). The correlations of 1,25D/25D ratios with other clinical parameters are summarized in Table 2. There were no significant correlations of 1,25D/25D with SDS of the patients' profile, blood cell count, liver function, coagulatory markers, inflammatory markers, and endocrine and metabolic markers (Table 2).

**Table 2:** Correlations between serum 1,25D/25D ratio and clinical parameters in all patients ACE: angiotensin-converting enzyme; ACTH: adrenocorticotropic hormone; ALP: alkaline phosphatase; ALT: alanine aminotransferase; APTT: activated partial thromboplastin time; AST: aspartate aminotransferase; BAP: bone-specific alkaline phosphatase; BMI: body mass index; BUN: blood urea nitrogen; CH50: 50% hemolytic unit of complement; Ca: calcium; cCa: corrected calcium; CRP: C-reactive protein; DEXA %YAM: dual-energy X-ray absorptiometry % young adult mean; eGFR: creatinine and estimated glomerular filtration rate; ESR: erythrocyte sedimentation rate in one hour; FSH: follicle-stimulating hormone; FT4: free thyroxine; γGTP: γ-glutamyl transpeptidase; GH: growth hormone; HbA1c: hemoglobin A1c; iP: inorganic phosphate; LDH: lactate dehydrogenase; LH: luteinizing hormone; PRL: prolactin; PTH: parathyroid hormone; PT-INR: prothrombin time-international normalized ratio; SDS: self-rating depression scale; TSH: thyroid-stimulating hormone; 25D: 25-hydroxyvitamin D; 1,25D: 1,25-dihydroxyvitamin D.

Comparison	All patients with 1,25D/25D		
Patients' profile	Number	R	P-values
Age	79	−0.10	0.43
BMI	79	0.26	*0.036
SDS	41	−0.11	0.56
Blood cell count			
White blood cell	78	−0.21	0.094
Red blood cell	78	0.16	0.21
Hemoglobin	78	0.098	0.45
Hematocrit	78	0.16	0.22
Platelet	78	−0.12	0.34
Bone metabolism			
Ca	78	0.18	0.16
cCa	77	0.17	0.18
iP	69	−0.34	**0.0081
cCa × iP	68	−0.24	0.065
ALP	36	0.46	*0.012
BAP	17	0.62	*0.0098
Intact PTH	30	0.64	**0.00029
Liver and renal functions			
Total bilirubin	68	0.00027	1.0
Total protein	70	0.059	0.66
Albumin	78	0.19	0.13
AST	74	−0.038	0.77
ALT	76	−0.046	0.72
LDH	75	−0.093	0.47
γGTP	74	−0.052	0.70
Sodium	79	0.020	0.88
Potassium	79	0.093	0.47
Chloride	79	0.15	0.25
Magnesium	57	0.27	0.063
BUN	78	−0.22	0.083
Creatinine	79	−0.26	*0.036
eGFR	78	0.11	0.38
Coagulatory markers			
PT-INR	38	−0.28	0.10
APTT	37	−0.24	0.16
D-dimer	30	−0.11	0.60
Inflammatory markers			
CRP	72	−0.24	0.067
ESR	29	−0.073	0.73
Ferritin	30	−0.12	0.56
CH50	22	−0.11	0.63
ACE	27	0.055	0.79
Endocrine and metabolic markers			
HbA1c	48	−0.089	0.58
Plasma glucose	55	0.24	0.12
Total cholesterol	61	0.16	0.26
Uric acid	72	−0.06	0.65
ACTH	30	−0.30	0.15
Cortisol	31	0.077	0.72
PRL	15	−0.40	0.18
LH	17	0.16	0.56
FSH	18	0.28	0.28
GH	18	−0.28	0.29
TSH	62	−0.12	0.40
FT4	60	−0.28	*0.047
TSH/FT4	60	0.042	0.77
Bone mineral density			
DEXA %YAM (Femoral)	49	−0.35	*0.023
DEXA %YAM (Lumbar)	49	−0.32	*0.039

Relevance of serum 1,25D/25D ratios to patients' age and BMI

**Figure 5:**
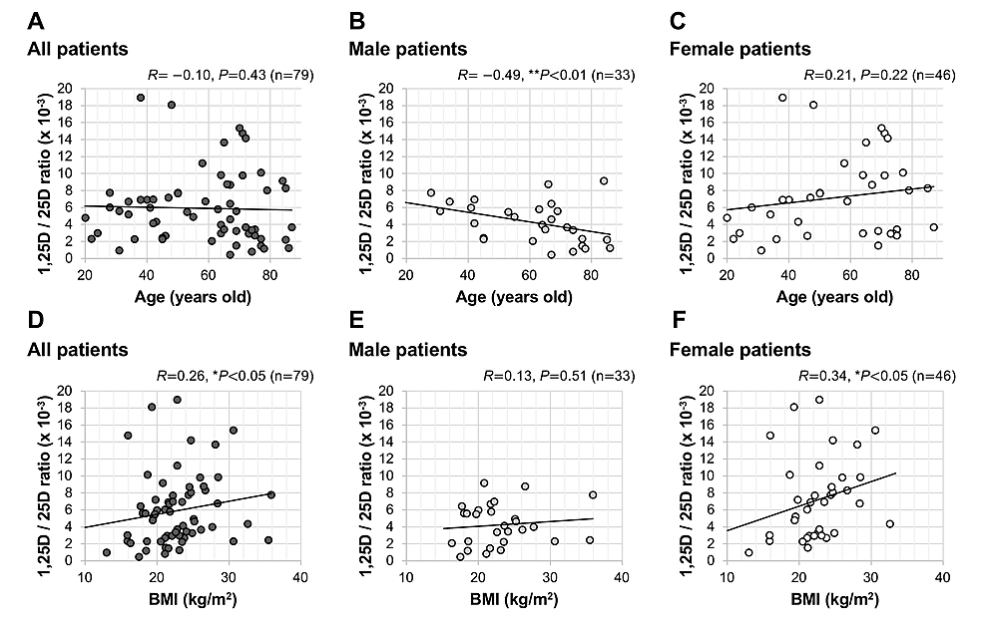
Correlations of patients' age and BMI in relation to serum 1,25D/25D ratio Correlations of age with serum 1,25D/25D ratio in all patients (A), male patients (B), and female patients (C) are shown. Correlations of BMI with serum 1,25D/25D ratio in all patients (D), male patients (E), and female patients (F) are also shown. **P<0.01 and *P<0.05, statistically significant correlations between the indicated factors. BMI: body mass index; 1,25D: 1,25-dihydroxyvitamin D; 25D: 25-hydroxyvitamin D.

1,25D/25D ratio did not show a significant correlation with age in all patients (*R*=−0.10, *P*=0.43; Figure 5A). However, when the patients were divided by gender, there was a significant correlation of 1,25D/25D ratio with age in male patients (*R*=−0.49, **P<0.01; Figure 5B), but there was no significant correlation in female patients (*R*=0.21, *P*=0.22; Figure 5C). 1,25D/25D ratio showed a significant positive correlation with BMI in all patients (*R*=0.26, *P<0.05; Figure 5D). When the patients were divided by gender, there was no significant correlation in male patients (*R*=0.13, *P*=0.51; Figure 5E), but there was a significant correlation in female patients (*R*=0.34, *P<0.05; Figure 5F).

## Discussion

The results of the present study suggested that vitamin D activities had relevance to clinical parameters, especially bone turnover, with gender-specific correlations with features in age and BMI. The ratio of serum 1,25D/25D as a marker for activation of vitamin D was significantly lower in male patients than in female patients, particularly in older females (≧50 years of age), who are considered to be menopausal women. On the other hand, bone mineral density was significantly lower in older female patients (≧50 years of age) than in male patients. The serum 1,25D/25D ratio was found to be negatively correlated with bone mineral density, negatively correlated with serum inorganic phosphate, and positively correlated with intact PTH, ALP, and BAP in all patients. Of interest, the ratio was negatively correlated with age in male patients but was positively correlated with BMI in female patients, suggesting that vitamin D activation is involved in bone metabolism in a gender-specific manner.

The 1,25D/25D ratio is a putative index of CYP27B1 activity and is considered to be a useful tool for the diagnosis of ocular sarcoidosis [17]. In cases of sarcoidosis or lymphomas, type II IFN enhances the activity of 1α-hydroxylase in macrophages, resulting in increased production of 1,25D and hypercalcemia [1]. Excessive vitamin D activity also has a stimulatory effect on bone turnover and an inhibitory effect on bone mineralization [18]. Vitamin D is a key component of the bone-kidney-parathyroid endocrine axis. 1,25D produced in the kidney binds to VDR in the bone and also activates FGF-23 gene expression. Secreted FGF-23 acts on the Klotho-FGF receptor complex in the kidney and parathyroid gland. In the kidney, FGF-23 down-regulates the *Cyp27b1* gene and up-regulates the *Cyp24* gene, resulting in suppression of vitamin D activity. In the parathyroid gland, FGF-23 suppresses the expression of PTH, which has the potential function of promoting *Cyp27b1* gene expression. Since there is a closed negative feedback loop for vitamin D homeostasis, disruption of the loop regulating CYP27B1 induction results in an increase in 1,25D level [19-21].

Vitamin D level in serum has been reported to decline with aging due to a reduction in the production of vitamin D in the skin [22,23]. In general, a hormonal decline of sex steroids such as androgen and estrogen is important in the aging process [24]. Total testosterone level has been reported to have a slight but significant positive association with serum 25D level, suggesting that both testosterone and vitamin D can be health-related markers for males [25]. A meta-analysis showed that vitamin D status has an inverse relationship with BMI in both diabetic and non-diabetic subjects [26]. Another meta-analysis showed that serum vitamin D level had an inverse association with the risk of abdominal obesity in a dose-response manner [27]. Vitamin D deficiency has been considered to be associated with obesity and metabolic dysregulation by modulating the expression of genes related to adipogenesis and inflammatory and oxidative stress in mature adipocytes [28].

In the present study, it was also shown that serum levels of creatinine and free thyroxin were negatively correlated with the serum 1,25D/25D ratio. In this regard, patients with chronic kidney disease (CKD) usually have secondary hyperparathyroidism and a low serum 1,25 level [29]. Patients in an advanced stage of CKD have high levels of serum FGF-23 and PTH and a low level of Klotho expression, so-called FGF-23 resistance, leading to impaired activation of vitamin D [19]. Vitamin D also acts on the thyroid through VDR; however, there is no clear consensus about a relationship between vitamin D status and thyroid function in healthy humans [30], although a study on the role of vitamin D in thyroid diseases indicated that vitamin D deficiency might be an increased risk of autoimmune thyroid diseases [30]. However, based on the present findings, it seems likely that thyroid function is involved in the activation of vitamin D.

Vitamin D activity should be evaluated when vitamin D-related disorders such as hyperparathyroidism or granuloma-forming disorders are suspected. However, our findings presented here indicate the importance of assessing vitamin D activity from the ratio of 1,25D to 25D in general clinical settings. Considering that vitamin D activation can be linked to aging and obesity as well as bone mineral metabolism, measurement of serum 1,25D/25D ratio can be useful for suspecting bone loss, fractures, sarcopenia, or other clinical outcomes associated with frailty. Since the present study showed a negative correlation between serum 1,25D/25D ratio and bone mineral density, serum 1,25D/25D ratio might be a marker for determining the necessity for vitamin D supplementation. However, when a high serum 1,25D/25D ratio is related to increased PTH as in primary hyperparathyroidism, vitamin D supplementation may promote the progression of hypercalcemia. Nevertheless, our findings suggest that a high serum 1,25D/25D ratio is a clue for considering the loss of bone mineral density. There are some limitations of the present study. Patients included in the present study had various pathological conditions possibly associated with hypovitaminosis D. Since we focused on BMI and age, which are physiological parameters potentially influenced by pathological conditions, our study could not show a direct interrelationship between vitamin D metabolism and BMI/age. However, we consider that it is meaningful to assess real-world data obtained from clinical practice in general medicine. Also, serum vitamin D levels can be affected by seasonal changes, lifestyles related to sunlight exposure, nutritional intake, and human race [5,23]. In the present study, serum vitamin D levels might have been affected by seasonal changes or sunlight exposure. All of the patients included in this study were Japanese.

Technically, although free vitamin D and albumin-bound vitamin D (10-15%) are bioavailable, current assays cannot distinguish free vitamin D from vitamin D-binding protein-bound (DPB) vitamin D (85-90%) and albumin-bound vitamin D, the amounts of which are affected by the capability for DPB and albumin synthesis [31]. Another limitation of this study is that it was performed retrospectively at a single center with a relatively small number of patients, and it is, therefore, difficult to draw a solid conclusion. To clarify the precise interaction between vitamin D activity and bone turnover, another study with a larger sample size including a general population or a prospective study using age- and gender-matched cohorts as a multi-center study will be needed.

In the present study, it was notable that the 1,25D/25D ratio is conceivably a useful tool for suspecting bone loss, fractures, or other clinical outcomes associated with frailty.

## Conclusions

The results of analysis of data for patients in our general medicine department collectively showed that increased activation of vitamin D is likely to be linked to disruption of the bone-kidney-parathyroid endocrine axis. Our findings imply the probable existence of a gender-specific difference of aging males and obese females regarding the activation of vitamin D that is functionally linked to bone metabolism.
